# Wing geometric morphometrics and *COI* barcoding of *Culex pipiens* subgroup in the Republic of Korea

**DOI:** 10.1038/s41598-024-51159-8

**Published:** 2024-01-09

**Authors:** Jiseung Jeon, Dong Yeol Lee, Yewon Jo, Jihun Ryu, Eunjeong Kim, Kwang Shik Choi

**Affiliations:** 1 School of Life Sciences, BK21 FOUR KNU Creative BioResearch Group, Kyungpook National University, Daegu, 41566 Republic of Korea; 2 School of Life Sciences, College of Natural Sciences, Kyungpook National University, Daegu, 41566 Republic of Korea; 3 Research Institute for Dok-do and Ulleung-do Island, Kyungpook National University, Daegu, 41566 Republic of Korea; 4 Research Institute for Phylogenomics and Evolution, Kyungpook National University, Daegu, 41566 Republic of Korea

**Keywords:** Evolution, Genetics

## Abstract

Two members of the *Culex pipiens* subgroup, *Culex pallens* and *Culex pipiens* f. *molestus*, are known to occur in the Republic of Korea (ROK). These species exhibit morphologically similar features and are challenging to distinguish below the species level. Therefore, this study utilized wing geometric morphometrics (GM) on the right wing of the *Culex pipiens* subgroup, alongside sequencing of the cytochrome *c* oxidase subunit I (*COI*) region. Mosquitoes were collected from 11 locations between June and October (2020–2022) to minimize regional and seasonal variations. Additionally, *Culex pipiens* f. *pipiens*, which is not native to the ROK, was included in the analysis. *Culex tritaeniorhynchus*, *Aedes albopictus*, and *Anopheles sinensis*, the primary vectors in the ROK, were used as outgroups for comparison. All three taxa in the *Culex pipiens* subgroup could be identified with an 82.4%–97.0% accuracy using GM. However, a comparison of the *COI* regions of the *Culex pipiens* subgroup revealed no clear differences between the taxa. These data can be used for accurate identification, contributing to effective mosquito control, in addition to providing a foundation for evolutionary and ecological studies on wing shape differences.

## Introduction

Despite decades of attempts to effectively control mosquitoes (Diptera: Culicidae), mosquito-borne diseases have proliferated globally, driven by rapid urbanization, climate change, and increased trade and travel^1,2^. Mosquito-borne diseases are primarily result from protozoan parasites and flaviviruses, which are transmitted when female mosquitoes feed on human or animal blood to acquire nutrients for oviposition^3^. Within various mosquito species, the *Culex pipiens* subgroup, prevalent near human habitation, stands out as a significant vector of the West Nile virus (WNV)^4–6^. Importantly, although no human cases of WNV have been reported in the Republic of Korea (ROK), its detection in domestic pigeons in 2015 highlights the need for vigilance^7^.

The *Culex pipiens* subgroup comprises five species (*Culex pipiens* Linnaeus, *Culex quinquefasciatus* Say, *Culex pallens* Coquillett, *Culex australicus* Dobrotworsky & Drummond, and *Culex globocoxitus* Dobrotworsky)^8–10^. Following the recent update in Culicidae nomenclature, there are no longer subspecies in mosquito classification. *Culex pipiens pallens* has been elevated to species status and is now referred to as *Culex pallens*^11^. *Culex pipiens* is recognized for having two ecological forms (*Culex pipiens* f. *pipiens* and *Culex pipiens* f. *molestus* Forskal)^8–10^. Among these taxa, *Cx. pallens* and *Cx. pipiens* f. *molestus* are currently identified in the ROK^12^. Despite their close evolutionary relationship, they exhibit distinct ecological differences^13^. *Cx. pallens* primarily targets birds (ornithophilic), engages in mating while in flight (eurygamy), and requires blood for oviposition (unautogeny)^14–16^. In contrast, *Cx. pipiens* f. *molestus* shows a preference for mammals (mammalophilic), mates in enclosed spaces (stenogamy), and does not require blood for the first spawn (autogeny)^13,17^. These species also differ in life cycles and vector competence^18,19^, which highlights the importance of precise identification for effective vector control. Nevertheless, due to their high morphological similarity, accurate classification remains challenging in the ROK^20^.

Geometric morphometrics (GM), a technique integrating geometric analysis and biology, facilitates the quantitative analysis of an organism’s features and size of an organism through statistical methods^21^. Therefore, this technique serves as a valuable complement to traditional taxonomic methods. The wings of mosquitoes are less prone to damage than scales or bristles and feature a two-dimensional vein structure that enables the straight forward collection of landmarks (LMs) for GM analysis. GM is also cost-effective and time-efficient^22^. While molecular markers are increasingly used for accurate identification of the *Culex pipiens* subgroup^23^, alternative methods are needed to compensate for the occasional limitations of molecular approaches. Additionally, GM has been actively employed in mosquito research, proving successful in various fields^24–26^. However, there is currently a severe lack of quantitative morphological analyses of pathogen vectors in the ROK.

Therefore, this study examined *Cx. pallens* and *Cx. pipiens* f. *molestus* of the *Culex pipiens* subgroup in the ROK using GM. Furthermore, *Cx. pipiens* f. *pipiens* of the *Culex pipiens* subgroup, which is absent in the ROK, was included in the analysis. Furthermore, major pathogen vectors in the ROK, including *Culex tritaeniorhynchus* Giles (the causative agent of Japanese encephalitis), *Aedes albopictus* Skuse (the causative agent of Zika), and *Anopheles sinensis* s.s. Wiedemann (the causative agent of Malaria), served as outgroups in the analysis^27–29^. Finally, sequencing of the DNA barcoding region [cytochrome *c* oxidase subunit I (*COI*)] was conducted to compare the results of the quantitative morphological analysis using GM.

## Methods

### Sample collection and identification

**Figure 1:**
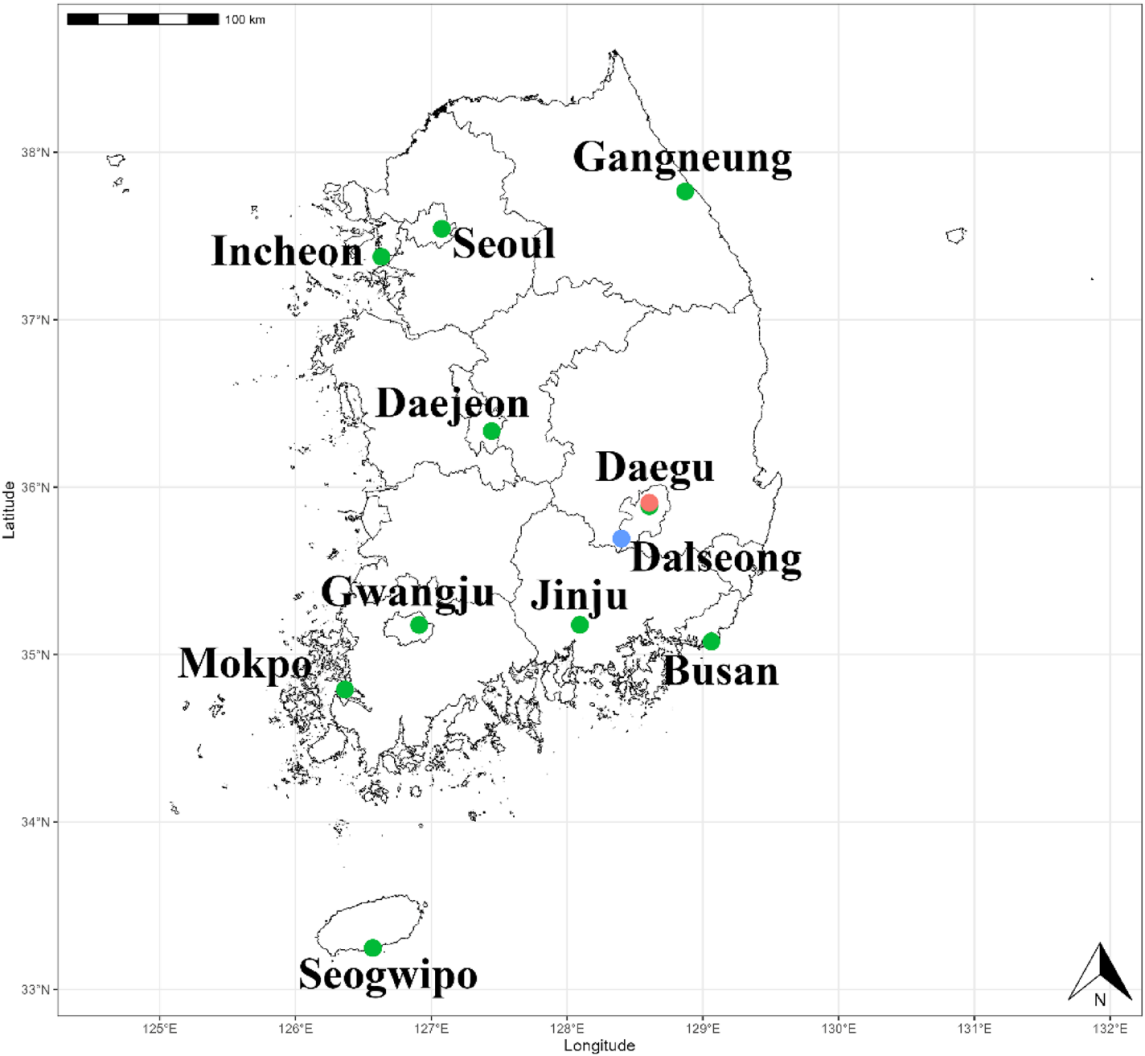
Sample collection sites in the ROK (*Culex pipiens* subgroup (green), *Ae. albopictus* (red), *Cx. tritaeniorhynchus* and *An. sinensis* (blue)). This map was generated using R v.4.2.1 (https://www.r-project.org/) and QGIS 3.26.3 (https://www.qgis.org/ko/site).

To eliminate regional and seasonal biases, specimens of the *Culex pipiens* subgroup were gathered from a total of 11 different sites in the ROK spanning from June to October (Fig. 1). The selected sampling sites were located around buildings in urban areas, taking into consideration the habitat preferences of the *Culex pipiens* subgroup BG-sentinel traps (Biogents, Regensburg, Germany) with dry ice were employed for collecting the *Culex pipiens* subgroup. Additionally, black light traps (BioTrap, Seoul, ROK) were deployed in a cow shed to capture *Cx. tritaeniorhynchus* and *An. sinensis*, which served as outgroups. As for *Ae. albopictus*, those captured alongside the *Culex pipiens* subgroup in Daegu were utilized. All captured mosquitoes were preserved at − 20 °C until identification.

The species within the *Culex pipiens* subgroup share a high morphological similarity, posing challenges for identification. Therefore, mosquito legs were detached from the bodies and utilized for identifying *Cx. pallens* and *Cx. pipiens* f. *molestus*, following the method described by Ryu and Choi^12^. Universal forward primers for *Cx. pallens* and *Cx. pipiens* f. *molestus* (F1457: 5′-GAG GAG ATG TGG AAT CCC AA-3′)^23^ were paired with species-specific reverse primers (ACEpall_R: 5′- ACA TGT CAA AAG CTC AGT TAG T -3′/ACEmole_R: 5′- TTC TCA CAG AGC CAT CAT CGA C -3′), as detailed by Ryu and Choi^12^ (Supplementary Fig. S1). Among the outgroup mosquitoes, *An. sinensis* was also typed to the species level using the molecular markers described by Bang et al.^30^. Universal forward primers for *Anopheles* species in the ROK (Universal forward primer: 5′- ATC GAT GAA GAC CGC AGC TA -3′) and eight species-specific reverse primers (*An. sinensis*: 5′- TAG GGT CAA GGC ATA CAG AAG G-3′/ *An. koreicus*: 5′- TAT CGT GGC CCT CGA CAG -3′/ *An. lindesayi*: 5′- ACC ATC TAC TGC CTG AAC GTG -3′/ *An. kleini*: 5′- TTT GTT GAT AAC TTG TAT CGT CCA TC -3′/ *An. lesteri*: 5′- CAG TCT CTT GCA GCC CAT TC-3′/ *An. sineroides*: 5′- CGC GCA CGC TCA GAT ATT -3′/ *An. belenrae*: 5′- TGT CCT AGG CGG TTA TCA ACA-3′/ *An. pullus*: 5′- CGG CGT AGT TTA TTG TGT ATA ACA TC-3′) were also used, as suggested by Bang et al.^30^. *Cx. tritaeniorhynchus* and *Ae. albopictus* can be clearly identified by morphological features and therefore require no molecular identification^31^. Table 1 provides information about the mosquitoes used in this experiment.

**Table 1:** Mosquitoes collected from 11 sites in the ROK between June and October.

Species	Collection sites											Total
	Seoul	Incheon	Gangneung	Daejeon	Mokpo	Gwangju	Jinju	Busan	Daegu	Dalseong	Seogwipo	
*Cx. pallens*		41	1		1		1		3			47
*Cx. pipiens* f.* molestus*	3	3		4		2	3	19	26		7	67
*Cx. tritaeniorhynchus*										45		45
*Ae. albopictus*									40			40
*An. sinensis*										35		35

### GM analysis

Only the right wings of the identified female mosquitoes were selectively used for the GM analysis. The right wing of each mosquito species was dissected, and wing scales were removed using a brush. Afterward, the wings were mounted on microscope slides and covered with coverslips using Canada balsam (Duksan, Seoul, ROK). These prepared wing specimens were photographed under 20 × magnification using an Olympus SZ61 Stereo Microscope (Olympus Corp., Tokyo, Japan). Wing images of *Cx. pipiens* f. *pipiens* collected in Germany, a species not distributed in the ROK, were incorporated into our experiments^32,33^.

**Figure 2:**
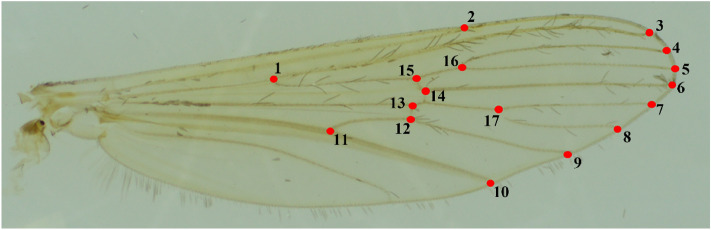
The 17 LMs set up on the right wing of a female adult mosquito for GM analysis.

Landmark coordinates were collected using TPSdig2 (2.31) (Fig. 2), and 17 LMs were established for the GM analysis of mosquito wings^34^. Beriotto et al.^35^ determined that 9, 13, and 17 LM settings produced the same total error rate in studies involving *Cx. pipiens*. Since this study involved three taxa of the *Culex pipiens* subgroup, 17 LMs were chosen for a more precise comparison. Subsequently, the TPS files for each mosquito taxon with established LMs were analyzed using various packages in R v.4.2.1^36^.

Procrustes analysis was conducted with the ‘geomorph’ package (v. 4.0.5) to transform the difference in position and direction of each data coordinate^37^. The results were superimposed to generate Procrustes coordinates. The adequacy of the established LMs was confirmed using the ‘LamBDA’ package (v. 0.1.1)^38^. Centroid size (CS), defined as the square root of the sum of the squared distances of all landmarks on the object from their centroid, was calculated from the Procrustes coordinates to estimate wing size^39^. The allometric effect, which represents the relationship between shape and size, was also evaluated using the ‘geomorph’ package (1000 permutations)^40^. Mean CS values for each species were compared using analysis of variance (ANOVA), and pairwise comparisons were conducted using t-tests with the ‘agricolae’ (v.1.3–7) package (Bonferroni adjusted p-value)^41^. To analyze differences in wing shape between groups, linear discriminant analysis (LDA) was performed using the ‘tidyverse’ (v.2.0.0) and ‘MASS’ (v.7.3–60) packages^42,43^. The convex hull algorithm, encompassing all points for each group in the scatterplot, was also applied. To compare the wing shape of each taxon, mean shapes for each LM coordinate were calculated and compared. The Mahalanobis distance, a statistical indicator of the difference between each group, was calculated using the ‘CVA’ function of the ‘Morpho’ package (v. 2.11) with 10,000 permutations^44^. Finally, a jackknife leave-one-out cross-validation was performed to determine the accuracy of the classification based on wing shape.

### *COI* barcoding

Genomic DNA was extracted from the whole body, except the right wing, of the species-identified mosquitoes using the Clear-S™ Quick DNA Extraction Kit (InVirusTech, Gwangju, ROK), following the manufacturer’s protocol.

Following genomic DNA extraction, universal primers (LCO1490: 5′-GGT CAA CAA ATC ATA AAG ATA TTG G-3′/HCO2198: 5′-TAA ACT TCA GGG TGA CCA AAA AAT CA-3′) were employed to amplify the *COI* region^45^. The polymerase chain reaction (PCR) amplification mixture (total: 25 μL) consisted of 1 μL of extracted genomic DNA, 1 × PCR buffer, 0.2 mM dNTPs, 1.5 mM MgCl_2_, 0.4 μL of each primer, and 0.5 units of *Taq* DNA polymerase (TaKaRa, Shiga, Japan). The PCR cycling conditions were as follows: initial denaturation at 94 °C for 5 min, followed by 30 cycles of 94 °C for 30 s, 56 °C for 30 s, 72 °C for 1 min, and a final extension at 72 °C for 5 min.

PCR amplification products were confirmed by electrophoresis, and samples with successful amplification were characterized via Sanger sequencing in both directions (Macrogen, Daejeon, Korea). The sequences were analyzed using BLAST, organized using the ‘msa’ (v. 1.28.0) package in R and Bioedit, and aligned using Clustal X^46–48^. The *COI* sequences obtained in this study were deposited in the NCBI GenBank database [accession numbers: OR835698 ~ OR835753]. The *COI* sequences of *Cx. pipiens* f. *pipiens* individuals were compared using sequences deposited in NCBI GenBank database (accession numbers: MT731275 ~ 6). For an accurate comparison, only the *COI* sequences of *Cx. pipiens* f. *pipiens* obtained by Sauer et al.^32^ used in this study were included in the phylogenetic analysis. The *COI* sequences (550 bp) were analyzed using the ‘ape’ (v. 5.7–1), ‘phangorn’ (v. 2.11.1), and ‘phytools’ (v. 1.5–1) packages^49–51^. A maximum likelihood (ML) tree was generated for phylogenetic analysis. After determining the nucleotide substitution model, GTR + I was identified as the best fit model. To assess the robustness of the tree, a bootstrap analysis with 1,000 replications was conducted. The ‘ggplot2’ (v. 3.4.2) and ‘ggtree’ packages (v. 3.4.4) were utilized to visualize the results of the phylogenetic analysis^52,53^.

## Results

### Wing GM and *COI* barcoding

The right wing samples for GM analysis consisted of 47 *Cx. pallens*, 67 *Cx. pipiens* f. *molestus*, 17 *Cx. pipiens* f. *pipiens*, 45 *Cx. tritaeniorhynchus*, 40 *Ae. albopictus*, and 35 *An. sinensis* samples (Table 1). To assess the sampling adequacy of LMs, we examined the LM sampling evaluation curve using the ‘LamBDA’ package, revealing no oversampling (fit = 0.90: 12 LMs; fit = 0.99: 16 LMs) (Supplementary Fig. S2).

**Figure 3:**
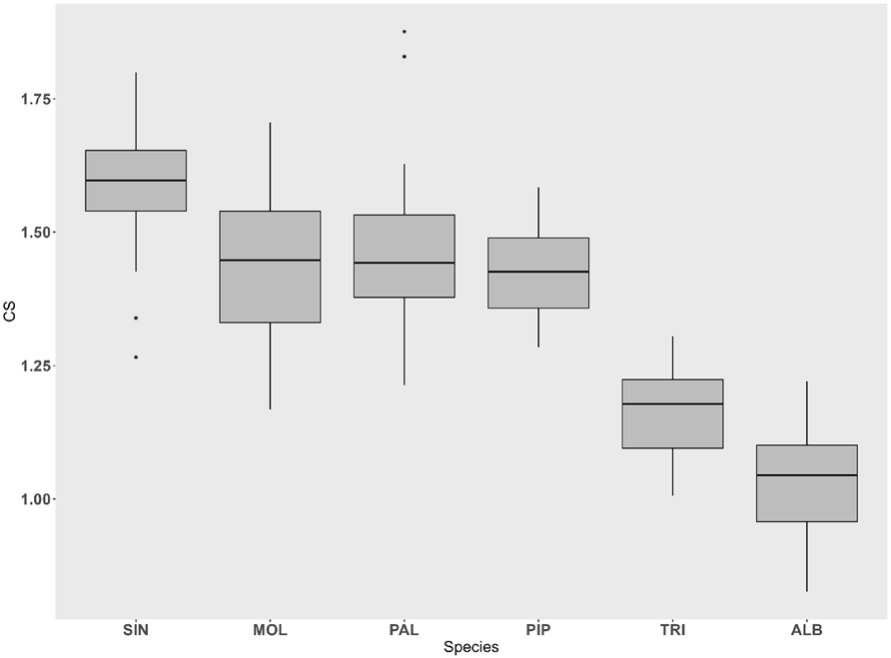
Boxplot showing the results of centroid size (CS) comparisons for each taxon (SIN = *An. sinensis*, PAL = *Cx. pallens*, MOL = *Cx. pipiens* f. *molestus*, PIP = *Cx. pipiens* f. *pipiens*, TRI = *Cx. tritaeniorhynchus*, and ALB = *Ae. albopictus*). The box represents the first and third quartiles, and the black line in the middle represents the median. The asterisks represent outliers.

The mean CS differed significantly between mosquito species (F = 125.7, p < 0.0001). On average, *An. sinensis* exhibited the largest CS, whereas *Ae. albopictus* had the smallest CS (Fig. 3). Pairwise comparisons of *Cx. pallens* vs. *Cx. pipiens f. molestus*, *Cx. pallens* vs. *Cx. pipiens* f. *pipiens*, and *Cx. pipiens* f. *molestus* vs. *Cx. pipiens* f. *pipiens* showed no statistically significant differences in CS (p = 1) (Supplementary Table S1). The allometric effect was found to be statistically significant (R^2^ = 3.3%, p < 0.0001), and it was analyzed together with the shape data, as it could provide useful information for the identification process^54,55^.

**Figure 4:**
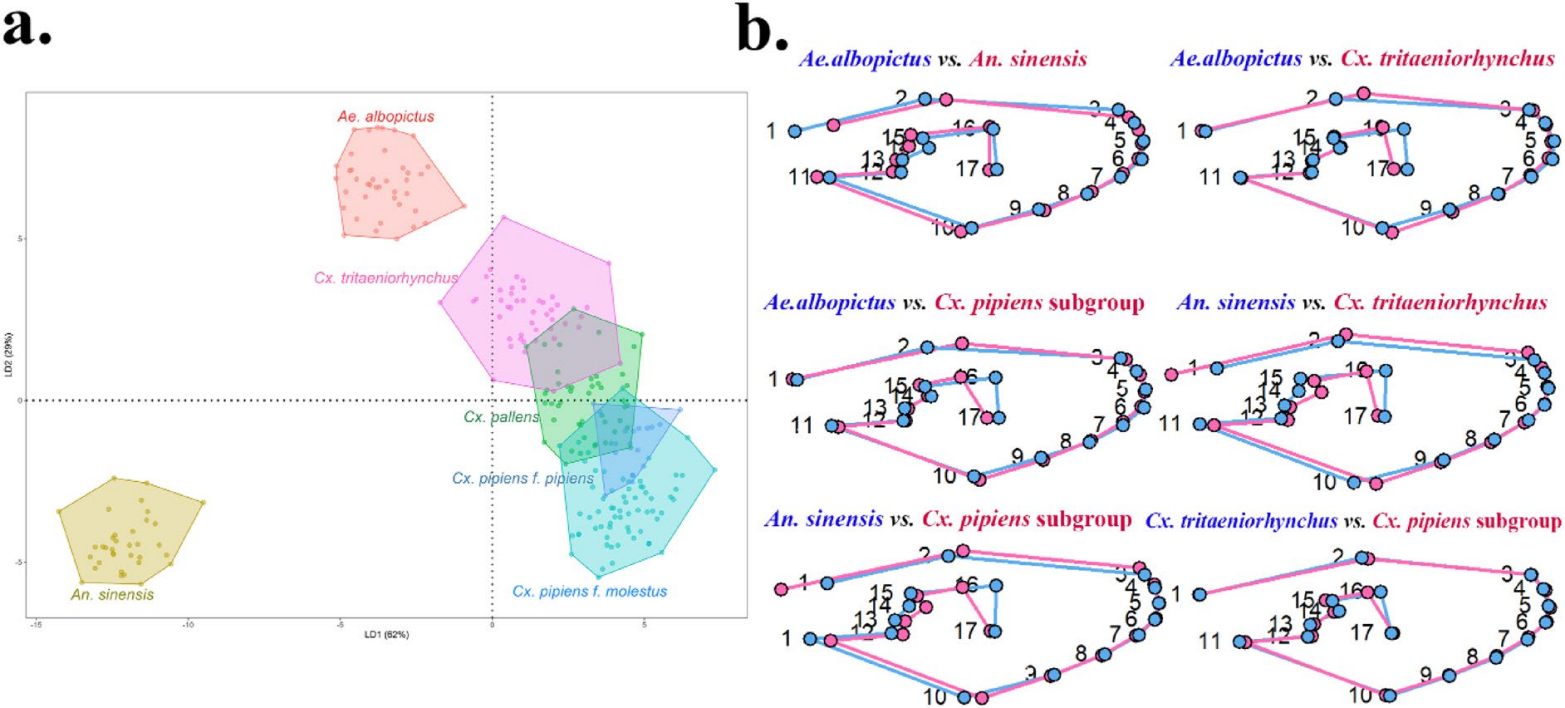
(**a**) Scatterplot of LDA results based on wing shape variation from the six taxa (LD1: 62%, LD2: 29%). (**b**) Comparison of mean wing shape for each taxon.

**Figure 5:**
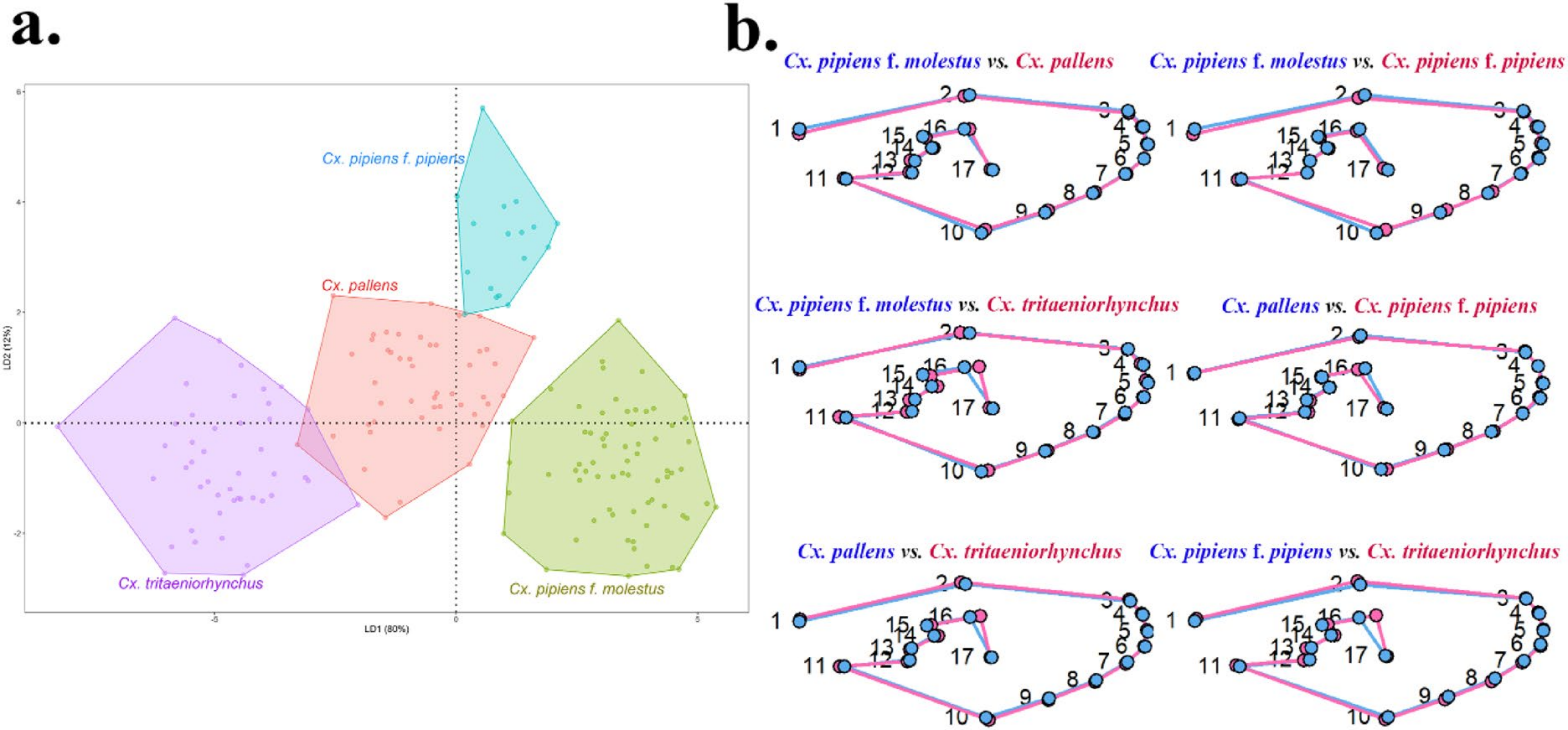
(**a**) Scatterplot of LDA results based on wing shape variation among species in the genus *Culex* (LD1: 80%, LD2: 12%). (**b**) Comparison of mean wing shape for each taxon.

Among the outgroup species*, An. sinensis* and *Ae. albopictus* were clearly separated, as shown in the LDA scatterplot results for all species analyzed together (Fig. 4a). *Cx. tritaeniorhynchu*s, also set as an outgroup, was generally well distinguished from mosquitoes in the *Culex pipiens* subgroup, with minor overlap in some specimens. Species within the genus *Culex pipiens* subgroup clustered together, displaying slight overlap for some species. Differences in mean wing shapes were observed in LM1-2 and LM16-17 for *An. sinensis* and *Ae. albopictus*, and mainly in LM1-2 and LM12-17 for *An. sinensis* and *Culex* mosquitoes (Fig. 4b). Significant differences in mean wing shape between *Ae. albopictus* and *Culex* mosquitoes were noted, particularly at LM2 and LM16-17. Subsequent LDA using only *Culex* mosquitoes revealed a clear separation of each taxon (Fig. 5a). A comparison of mean wing shapes within the genus *Culex* (Fig. 5b) indicated no major differences in wing shape for each taxon, with some distinctions primarily in LM16-17. The Mahalanobis distance calculated using canonical variate analysis showed that the closest taxa were *Cx. pallens* and *Cx. pipiens* f. *pipiens* (4.3152, p < 0.0001), followed by *Cx. pallens* and *Cx. tritaenioryhnchus* (4.4569, p < 0.0001). The most dissimilar species were *An. sinensis* and *Cx. pipiens* f. *pipiens* (17.2230, p < 0.0001) (Supplementary Table S2). Cross-validation (leaving-one-out method) for classification accuracy showed an overall average accuracy of 96.8% (Table 2). The outgroups *An. sinensis* and *Ae. albopictus* achieved 100% (*An. sinensis,* 35/35; *Ae. albopictus,* 40/40) accuracy, whereas *Cx. tritaeniorhynchus* achieved 97.7% (44/45) accuracy. Within the *Cx. pipiens* subgroup, *Cx. pallens* achieved 95.8% (45/47) accuracy, *Cx. pipiens* f. *molestus* achieved 97.0% (65/67), and *Cx. pipiens* f. *pipiens* achieved 82.4% (14/17), with the lowest accuracy, resulting in many individuals being misidentified as *Cx. pallens* (3/17).

**Table 2:** Cross-validation (leaving-one-out method) test results for six taxa based on wing shape (overall accuracy = 96.8%).

	*An. sinensis*	*Ae. albopictus*	*Cx. tritaeniorhynchus*	*Cx. pipiens* f. *molestus*	*Cx. pipiens* f*. pipiens*	*Cx. pallens*
*An. sinensis*	100.0% (35/35)	0%	0%	0%	0%	0%
*Ae. albopictus*	0%	100.0% (40/40)	0%	0%	0%	0%
*Cx. tritaeniorhynchus*	0%	0%	97.7% (44/45)	0%	0%	2.3% (1/44)
*Cx. pipiens* f. *molestus*	0%	0%	0%	97.0% (65/67)	0%	3.0% (2/65)
*Cx. pipiens* f.* pipiens*	0%	0%	0%	0%	82.4% (14/17)	17.6% (3/17)
*Cx. pallens*	0%	0%	0%	2.1% (1/47)	2.1% (1/47)	95.8% (45/47)

**Figure 6:**
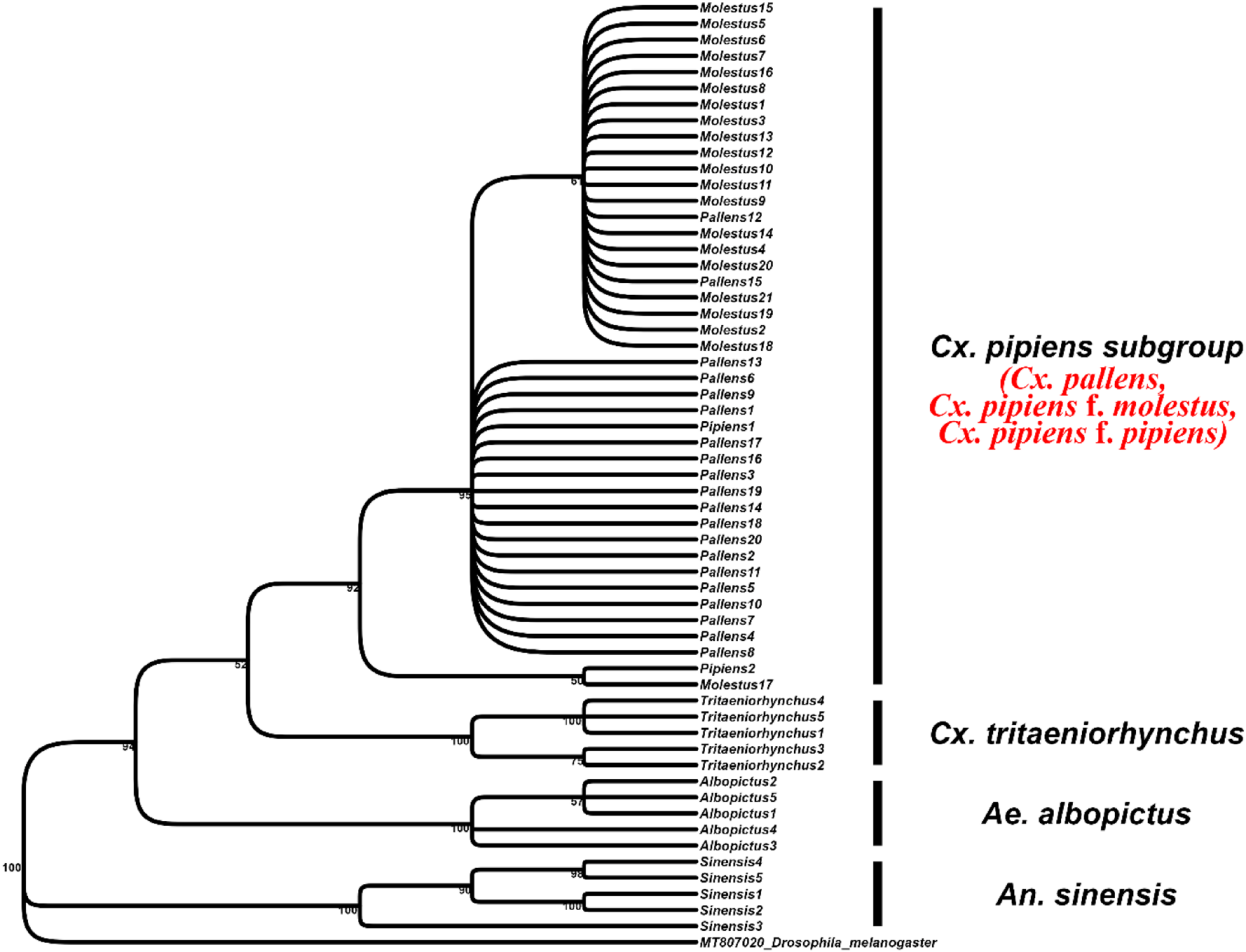
Maximum likelihood (ML) phylogenetic tree (GTR-I). The bootstrap value is shown next to each node (1,000 replicates). Unresolved taxa are shown in red text. *Drosophila melanogaster* (GenBank accession number: MT807020) was used as the outgroup.

As shown in the ML tree, *An. sinensis*, *Ae. albopictus*, and *Cx. tritaeniorhynchus*, designated as outgroups, formed a monophyletic clade with strong bootstrap support. However, species within the *Cx. pipiens* subgroup were unresolved (Fig. 6).

## Discussion

*Cx. pipiens* f. *molestus* and *Cx. pallens* differ in the D/V ratio of male genitalia and ommatidial number of females^56^. However employing such methods for identification is challenging for inexperienced researchers, time-consuming, and expensive, particularly when dealing with large numbers of individuals. Molecular biological approaches serve as valuable tools to overcome the challenges of morphological identification^23,57,58^ and remain the most powerful methods for accurate species identification. In response to these challenges, the analysis of mosquito wings using GM has gained acceptance and has proven to be a reliable and efficient method, complementing traditional morphological classification methods^24,32,54,55^. This study compared three taxa within the *Culex pipiens* subgroup (Cx. *pipiens* f. *molestus*, *Cx. pipiens* f. *pipiens*, and *Cx. pallens*) with *Cx. tritaeniorhynchus*, *Ae. albopictus*, and *An. sinensis* as outgroups. The results demonstrated that species identification using GM can be successfully applied not only at the level of evolutionarily distant genera but also in very closely related relationships. However, cross-validation results indicated that reliable classification of *Cx. pipiens* f. *pipiens* could not be achieved with high accuracy (14/17:82.4%). For example, *Cx. pipiens* f. *pipiens* and *Cx. torrentium*, known to co-occur sympatrically in Europe, cannot be easily differentiated via traditional morphological approaches, whereas GM was able to successfully discriminate them^59^. There are also examples of high heterogeneity in spatially separated populations of the same species^60^. However, in the case of *Cx. pipiens* f. *pipiens* used in this study^32,33^, it was not clearly identified using GM, despite its absence in the ROK and geographical separation. Therefore, further studies with larger geographic sampling are necessary. Nevertheless, only *Cx. pipiens* f. *molestus* and *Cx. pallens* of the *Culex pipiens* subgroup are currently known to occur in the ROK. GM-based methods are thus expected to be successful in identifying various pathogen vector mosquitoes, including the *Culex pipiens* subgroup in the ROK. Analyzing wing size using CS as an indicator revealed no statistically significant differences in wing size among the three taxa of the *Culex pipiens* subgroup. Furthermore, wing size in insects, including mosquitoes, is strongly influenced by the environment^61^, suggesting that using wing size for discrimination is not suitable for classifying close relatives like the *Culex pipiens* subgroup. However, LDA based on wing shape variation demonstrated that wing vein structure is homologous, species-specific, and less influenced by the environment than wing size. This makes it an extremely useful morphological trait for species identification, phylogenetic analysis, and tracking evolutionary relationships^62^.

In this study, the analysis of the *Culex pipiens* subgroup was based on data from *Cx. pallens* and *Cx. pipiens* f*. molestus* from the ROK and *Cx. pipiens* f. *pipiens* from Germany^32,33^. While *Cx. quinquefasciatus*, another member of the *Culex pipiens* subgroup, was not included in this experiment, its wide distribution in tropical regions and tendency to co-occur with other subgroup members suggests the need for further experiments to determine the feasibility of accurate species identification using GM compared to other members of the *Culex pipiens* subgroup^8,16,63^. As suggested by Dujardin et al.^64^, the wing images used in this study will be made available through an open-access repository, allowing researchers to use them in their own studies (10.5061/dryad.gtht76ht6).

The sequences of the *COI* region of *Cx. pallens* and *Cx. pipiens* f. *molestus* from the ROK was compared with that of *Cx. pipiens* f. *pipiens*, but no clear differences were observed. The *COI* region is commonly used for accurate species delimitation in insects and identifying genetic divergence in closely related species, and it has been extensively successful^65^. In the case of the *Cx. pipiens* subgroup, the influence of *Wolbachia* infection on mitochondrial diversity and the occurrence of sympatric populations capable of hybridization may contribute to limited genetic variation in the *COI* region. Thus, future comparisons between populations should adopt a multi-loci approach, considering both mitochondrial and nuclear genes^12,66,67^.

In the ROK, despite the *Culex pipiens* subgroup being a diverse pathogen vector, its accurate identification been challenging due to difficulties in morphological classification^5,20,68^. The successful use of GM in classifying the *Culex pipiens* subgroup, along with *Cx. tritaeniorhynchus*, *Ae. albopictus*, and *An. sinensis*, suggests the potential applicability of GM-based classification to various mosquito species in the ROK. This method could benefit researchers unfamiliar with mosquito taxonomy and those facing challenges with molecular biological approaches, providing a means for accurate species identification. Furthermore, GM analysis is not confined to interspecific variation and has been applied in various studies, including sexual dimorphism and parasite detection^69,70^. Therefore, the results of this study can serve as foundational data for future ecological and evolutionary research on the *Culex pipiens* subgroup. The wing images utilized in this study are also expected to be valuable resources for researchers in various fields.

### Supplementary Information

Supplementary Information.

## Data Availability

Sequencing data used in this study are deposited in the NCBI GenBank database (https://www.ncbi.nlm.nih.gov/) under the accession numbers (OR835698 ~ OR835753). The wing images used in this study are available at 10.5061/dryad.gtht76ht6.
