# Supplementary material for: Wing geometric morphometrics and COI barcoding of Culex pipiens subgroup in the Republic of Korea
Source: Sci Rep. 2024 Jan 9;14:878. doi: 10.1038/s41598-024-51159-8 (PMC10776869; doi:10.1038/s41598-024-51159-8)
Supplement: Supplementary file 1 — Supplementary Information. [file 41598_2024_51159_MOESM1_ESM.docx]

**Wing geometric morphometrics and COI barcoding of *Culex pipiens* subgroup in the Republic of Korea**

Jiseung Jeon^1,2,3^, Dong Yeol Lee^2^, Yewon Jo^2^, Jihun Ryu^1,2,3^, Eunjeong Kim^2^ and Kwang Shik Choi^1,2,3,4*^

**^1^ School of Life Sciences, BK21 FOUR KNU Creative BioResearch Group, Kyungpook National University, Daegu 41566, Republic of Korea**

^2^ School of Life Sciences, College of Natural Sciences, Kyungpook National University, Daegu 41566, **Republic of Korea**

^3^ Research Institute for Dok-do and Ulleung-do Island, Kyungpook National University, Daegu 41566, **Republic of Korea**

^4^ Research Institute for Phylogenomics and Evolution, Kyungpook National University, Daegu 41566, **Republic of Korea**

***Correspondence:** *ksc@knu.ac.kr*

School of Life Sciences, Kyungpook National University, Daegu 41566, **Republic of Korea**

Tel: +82-53-950-5351, Fax: +82-53-953-3066

**Supplementary materials**

**Supplementary Figure 1** Multiplex PCR assay for the identification of *Cx. pipiens* f. *molestus* and *Cx. pallens* in the *Culex pipiens* subgroup. Lane M, 1:1500 bp molecular marker; Lane 1~5, *Cx. pipiens* f. *molestus*; Lane 6~10, *Cx. pallens*; Lane N, Negative control.

**Supplementary Figure 2** Landmark sampling evaluation curve. Each gray line represents a single iteration, with a total of 1,000 iterations. The dark line is the average value (fit = 0.90: 12 LMs; fit = 0.99: 16 LMs).

**Supplementary Table 1** P-values of the pairwise comparisons for the CS of each mosquito taxon. The calculations were validated using t-tests and p-values were adjusted via Bonferroni correction.

**Supplementary Table 2** Mahalanobis distance between each taxon**.**

**
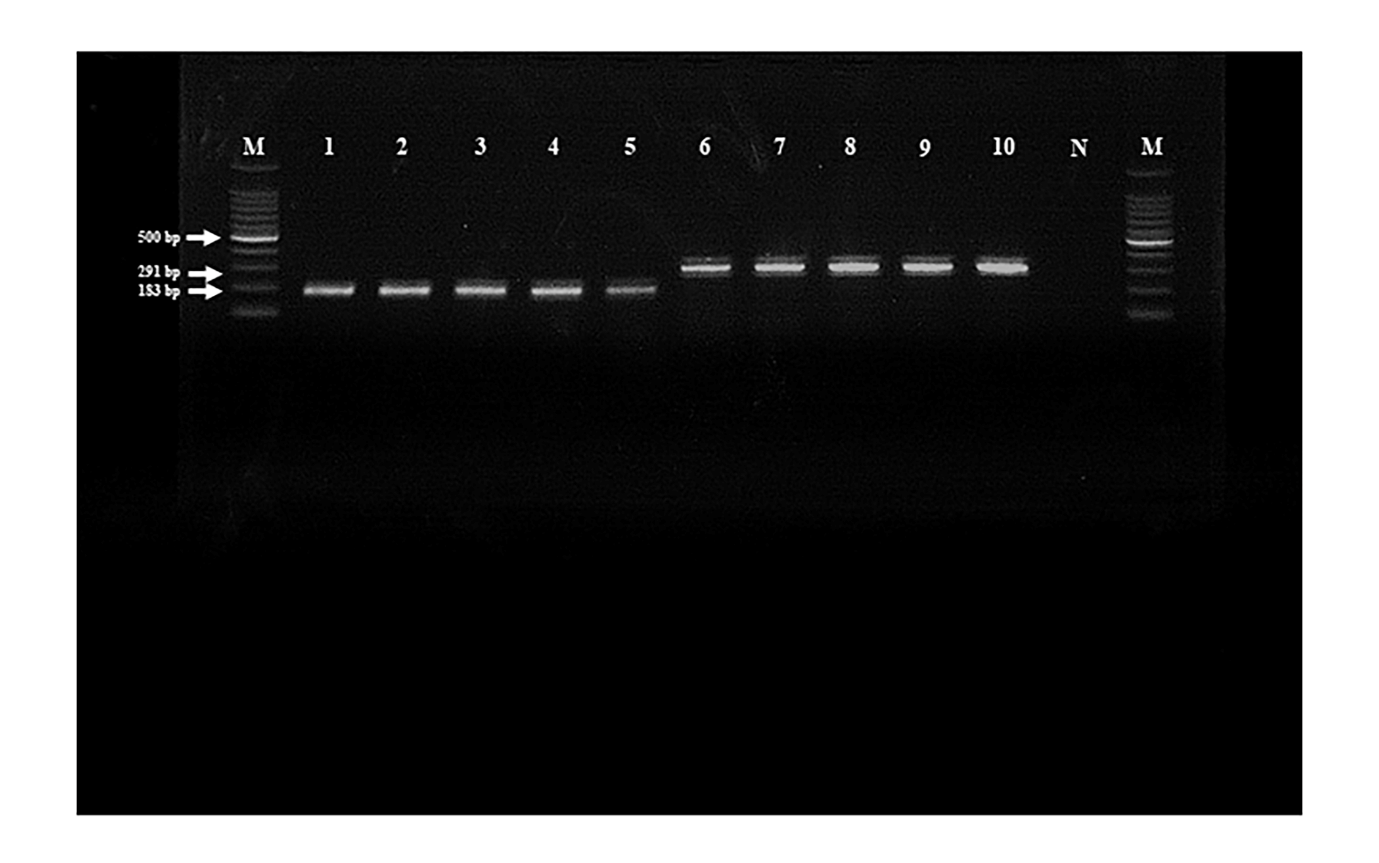
**

**Figure S1.** Multiplex PCR assay for the identification of *Cx. pipiens* f. *molestus* and *Cx. pallens* in the *Culex pipiens* subgroup. Lane M, 1:1500 bp molecular marker; Lane 1~5, *Cx. pipiens* f. *molestus*; Lane 6~10, *Cx. pallens*; Lane N, Negative control.

**
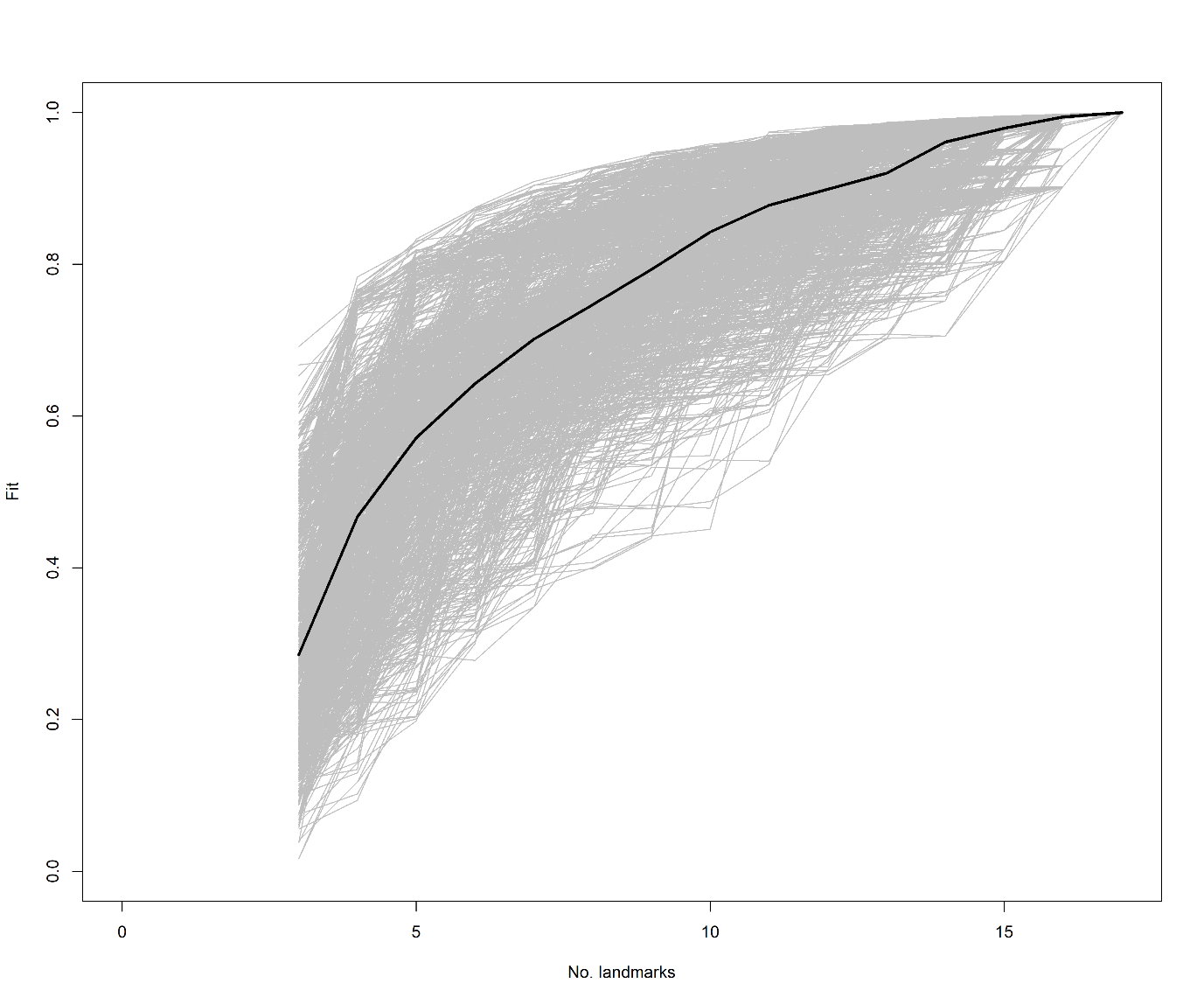
**

**Figure S2.** Landmark sampling evaluation curve. Each gray line represents a single iteration, with a total of 1,000 iterations. The dark line is the average value (fit = 0.90: 12 LMs; fit = 0.99: 16 LMs).

**Table S1. P-values of the pairwise comparisons for the CS of each mosquito taxon. The calculations were validated using t-tests and p-values were adjusted via Bonferroni correction.**

|  | ***Cx. pipiens* f. *molestus*** | ***Cx. pipiens* f. *pipiens*** | ***Cx. pallens*** | ***Cx. tritaeniorhynchus*** | ***Ae. albopictus*** |
| --- | --- | --- | --- | --- | --- |
| ***Cx. pipiens* f. *pipiens*** | **1.000** |  |  |  |  |
| ***Cx. pallens*** | **1.000** | **1.000** |  |  |  |
| ***Cx. tritaeniorhynchus*** | **<0.001** | **<0.001** | **<0.001** |  |  |
| ***Ae. albopictus*** | **<0.001** | **<0.001** | **<0.001** | **<0.001** |  |
| ***An. sinensis*** | **<0.001** | **<0.001** | **<0.001** | **<0.001** | **<0.001** |

**Table S2. Mahalanobis distance between each taxon.**

|  | ***Cx. pipiens* f. *molestus*** | ***Cx. pipiens* f. *pipiens*** | ***Cx. pallens*** | ***Cx. tritaeniorhynchus*** | ***Ae. albopictus*** |
| --- | --- | --- | --- | --- | --- |
| ***Cx. pipiens* f. *pipiens*** | **4.6683** |  |  |  |  |
| ***Cx. pallens*** | **4.8521** | **4.3152** |  |  |  |
| ***Cx. tritaeniorhynchus*** | **8.0181** | **6.7192** | **4.4569** |  |  |
| ***Ae. albopictus*** | **12.7784** | **12.1832** | **10.1589** | **8.0681** |  |
| ***An. sinensis*** | **16.5842** | **17.2230** | **15.8679** | **15.2674** | **14.2407** |
